# Room Temperature Viscous Flow of Amorphous Silica Induced by Electron Beam Irradiation

**DOI:** 10.1002/advs.202205237

**Published:** 2023-01-13

**Authors:** Sebastian Bruns, Christian Minnert, Laszlo Pethö, Johann Michler, Karsten Durst

**Affiliations:** ^1^ Department of Materials Science Technical University of Darmstadt Alarich‐Weiss‐Straße 2 DE‐64287 Darmstadt Germany; ^2^ Empa Swiss Federal Laboratories for Materials Science and Technology Feuerwerkerstrasse 39 Thun CH‐3602 Switzerland

**Keywords:** amorphous silica, electron beam irradiation, high temperature testing, micropillar compression, nanoindentation, viscosity

## Abstract

The increasing use of oxide glasses in high‐tech applications illustrates the demand of novel engineering techniques on nano‐ and microscale. Due to the high viscosity of oxide glasses at room temperature, shaping operations are usually performed at temperatures close or beyond the point of glass transition *T*_g_. Those treatments, however, are global and affect the whole component. It is known from the literature that electron irradiation facilitates the viscous flow of amorphous silica near room temperature for nanoscale components. At the micrometer scale, however, a comprehensive study on this topic is still pending. In the present study, electron irradiation inducing viscous flow at room temperature is observed using a micropillar compression approach and amorphous silica as a model system. A comparison to high temperature yielding up to a temperature of 1100 °C demonstrates that even moderate electron irradiation resembles the mechanical response of 600 °C and beyond. As an extreme case, a yield strength as low as 300 MPa is observed with a viscosity indicating that *T*_g_ has been passed. Those results show that electron irradiation‐facilitated viscous flow is not limited to the nanoscale which offers great potential for local microengineering.

## Graphical abstract

The electron gun of a scanning electron microscope is used to induce room temperature viscous flow in amorphous silica glass during micropillar compression. An accompanied ultra‐high temperature study reveals moderate electron irradiation to resemble a material condition usually found at temperatures further than 600 °C. Strong electron irradiation even induces a viscosity expected to be found at temperatures beyond *T*_g_.

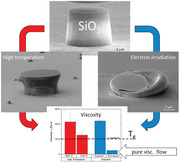

## 1 Introduction

Even though oxide glasses are often considered as prototype brittle material they became important structural and functional members of nowadays micro and nano components. Their application ranges from optics, over microelectromechanical systems (MEMS) to data storage where they are used in the forms of nanowires, thin films, pillars, or particles; just to name a view.^[^ ^1^, ^2^, ^3^, ^4^ ^]^ However, their brittle nature makes glass components susceptible to failure and presents difficulties in synthesis and assembly. This behavior is due to the simply too low contribution of viscous flow to the accommodation of external deformation rates at room temperature (RT), even though the opportunity to perform local forming operations would be highly desirable for various micro and nano applications.^[^ ^4^ ^]^ Instead, glass forming operations are usually performed at temperatures beyond the glass transition temperature *T*_g_, i.e., 1480 K for amorphous silica at normal pressure.^[^ ^5^, ^6^, ^7^ ^]^ At these temperatures, the threshold viscosity *η* of 10^12^ Pas is undercut and pure viscous flow is expected to occur.^[^ ^8^, ^9^, ^10^, ^11^ ^]^

In the past decades, a couple of theories were introduced to describe the viscous flow of glasses or amorphous systems in general. Doremus provides a comprehensive overview of those theories concluding network defect‐based theories to be the best promise to explain viscous flow in amorphous silica.^[^ ^12^ ^]^ A name closely associated with defect‐based concepts of viscous flow in oxide glasses is Mott, who proposed in the late 1980s that viscous flow is based on a bond‐switch mechanism.^[^ ^13^ ^]^ According to his concept viscous flow can be imagined as the motion of (point) defects throughout the silica network. The defects create a three‐fold coordinated silicon atom with a nonbridging oxygen (NBO) atom resulting from a broken siloxane (–Si—O–Si–) bond. Those so‐called dangling bonds reform with other neighboring atoms as it has meanwhile been confirmed by molecular dynamics (MD) simulations.^[^ ^4^, ^14^ ^]^ Upon bond‐switching atomic clusters (silicon monoxide molecules) rotate and migrate and can even align in dislocation‐like line defects. Those (not necessarily straight) line defects provide for a more efficient viscous flow compared to considering point defects only.^[^ ^12^, ^15^ ^]^ However, the flexibility of individual silicon monoxide molecules depends strongly on the neighboring bond strength distribution. Defects or for instance other ions are capable to induce bond strength fluctuations.^[^ ^12^ ^]^ Hence, a glass can be imagined exhibiting a structural heterogeneity of more and less rigid regions which govern viscous flow.^[^ ^16^, ^17^ ^]^ According to the topological constraint theory the viscosity scales with the number of atomic degrees of freedom in a glass network in an inverse manner.^[^ ^18^, ^19^ ^]^

At room temperature, stable viscous flow in oxide glasses is reported to be limited to the nanoscale. Extreme plasticity has been observed for amorphous silica nanospheres or ‐wires where the surface‐to‐volume ratio is beneficial to trigger viscous flow due to a large number of surface atoms with defective siloxane bonds present.^[^ ^4^, ^14^, ^20^, ^21^, ^22^ ^]^ At this scale, the electron irradiation present within the scanning‐ or transmission electron microscope (SEM/TEM) was found to induce a further softening of amorphous silica.^[^ ^4^ ^]^ It is well known that electron irradiation may alter structure and properties of a specimen.^[^ ^23^ ^]^ In the case of amorphous silica, MD simulations revealed two damage mechanisms. First, electron irradiation induces ionizing damage resulting in broken siloxane bonds. Second, intense electron irradiation is able to displace a bridging oxygen atom to form an interstitial O_2_ molecule leaving behind a vacancy.^[^ ^4^, ^24^, ^25^ ^]^ Latter process is expected to take place at high acceleration voltages in the range of 200 kV as found in TEM while the first process is expected to occur at acceleration voltages below 30 kV as found in conventional SEM.^[^ ^4^, ^26^ ^]^ Those defects induce additional degrees of freedom which reduce the flow stress and mediate very large ductility, the authors term as superplastic.^[^ ^4^ ^]^ Recently, the first attempts to use this effect productively for shaping have been described.^[^ ^26^ ^]^ Moreover, electron beam‐induced densification, changes in electrical conductivity and enhancements of fracture properties are reported in the literature.^[^ ^27^, ^28^, ^29^ ^]^

Electron irradiation effects on the viscous flow behavior of oxide glasses are well studied on the nanoscale. In those studies, the examined geometries range from 30 nm (nanowire) to 500 nm (nanosphere) in diameter.^[^ ^4^, ^26^, ^28^, ^29^ ^]^ On the micron scale, however, there is to the best of the authors knowledge no such data available. In a recent study fracture enhancement of 200% and beyond was reported in pillar‐splitting experiments on micron‐scale amorphous silica (Ø ≈ 5 µm) once the experiment was performed under running electron beam condition.^[^ ^27^ ^]^ Those results indicate that electron irradiation induces serious changes to micron‐scale amorphous silica as well. The present study aims to characterize the viscous flow behavior of micron‐scale amorphous silica under successively increasing electron irradiation intensity using a micropillar compression approach. The study is accompanied by high‐temperature micropillar compression experiments up to 1100 °C, a temperature slightly below *T*_g_, which allows for a correlation of electron irradiation‐induced viscous flow to high‐temperature thermally induced viscous flow.

## 2 Electron Irradiation Induced Softening of Amorphous Silica

**Figure 1:**
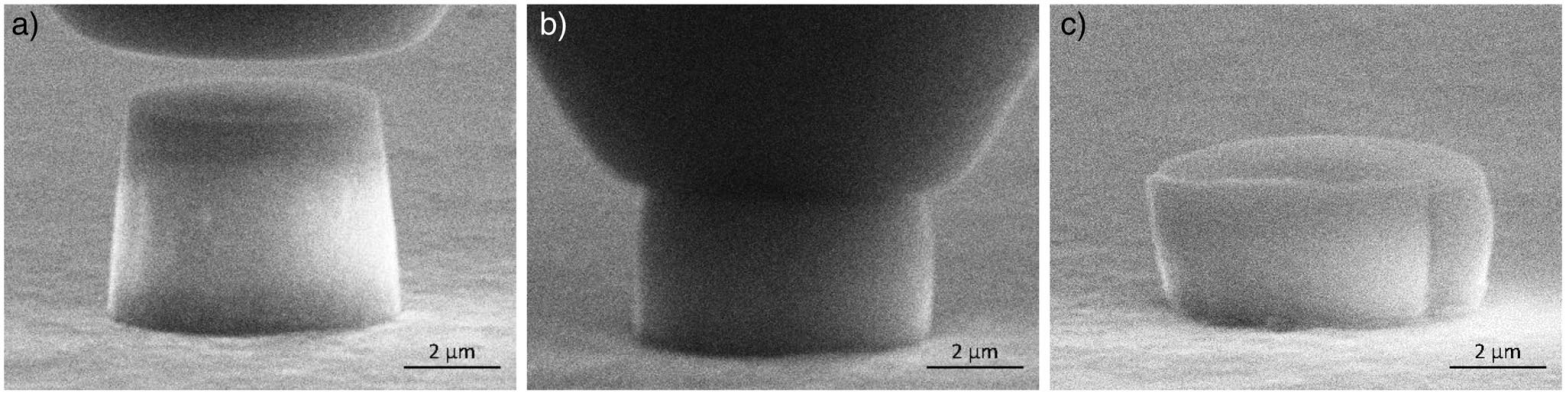
Micro pillar compression under a 20 kV and 11 A m^−^^2^ electron beam at three stages: a) before, b) during and c) after compression.

The micropillar compression experiments were performed inside the scanning electron microscope (SEM) as portrayed in **Figure** **1**. The 7 µm diameter diamond indenter tip is aligned centered above the amorphous silica micropillars with a diameter in the range of 5 µm (Figure 1a). During the compression experiment, the tip is moving downwards, loading and deforming the micropillar (Figure 1b), before it is retracted to release the deformed micropillar (Figure 1c).

**Figure 2:**
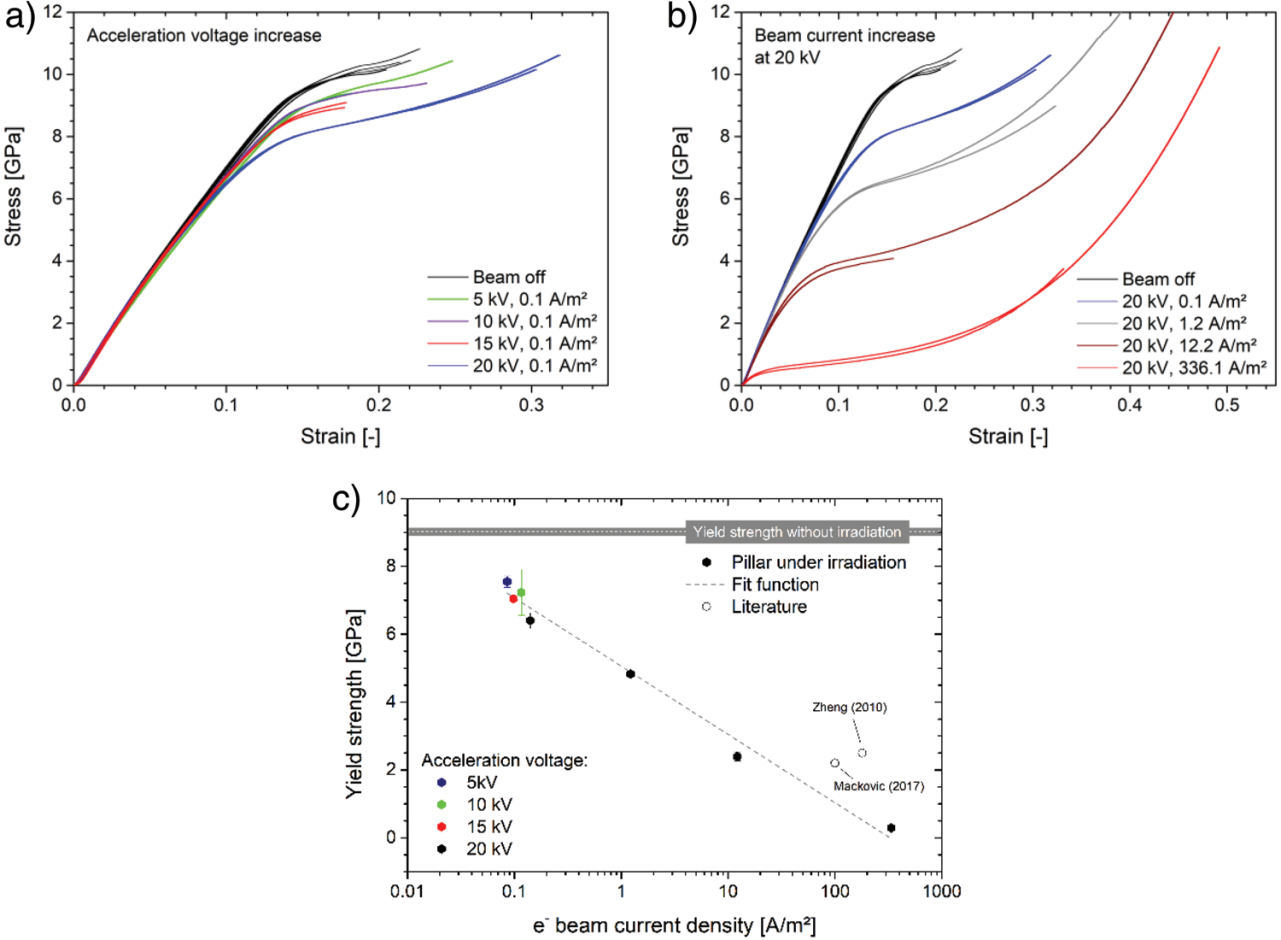
The stress as a function of the strain upon micropillar compression for a) increasing acceleration voltage at low beam currents and b) increasing electron beam densities at a constant acceleration voltage of 20 kV. c) The yield strength decays exponentially with increasing acceleration voltage and electron beam density (log scale). Literature data is shown as open symbols.^[^ ^4^, ^22^ ^]^

**Figure 3:**
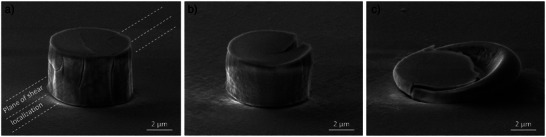
Micro pillar condition after the compression experiments. The deformation behavior of vitreous silica alters from a) brittle (beam off condition) over b) some intermediate state (5 kV, 0.1 A m^−^^2^) to c) ductile upon electron beam irradiation (20 kV, 336 A m^−^^2^ irradiation).

The recorded stress‐strain curves obtained from the compression experiments are presented in **Figure** **2**a. The reference curve (black) was recorded in a beam‐off condition of the SEM. It is worth to mention, however, that a weak irradiation during micropillar to indenter positioning had been unavoidable before testing. All following experiments were conducted under electron irradiation, starting with a configuration usually used for imaging amorphous silica in the SEM (5 kV, 0.1 A m^−^^2^). Interestingly, this beam configuration already shows a significant effect on the mechanic response of amorphous silica, proofing amorphous silica to be highly susceptible toward electron beam irradiation, even on the micron‐scale. The yield strength is reduced from ≈9 GPa to roughly 7.5 GPa, which corresponds to a drop of ≈15% (Figure 2c). A glance at the deformed micro pillar reveals that the initial taper angle has been compensated to a great extent by plastic deformation. A shear localization accompanied by brittle failure is observed for beam‐off condition (**Figure** **3**a). Steps, which may be interpreted as cracks, are visible at points where the shear localization interacts with a free surface, i.e., there are two steps visible on the micro pillars top surface and one at the left side of the micropillar at roughly 2/3^rd^ height. Moreover, regularly spaced radial cracks can be found along the micropillar periphery. Such type of cracks have already been reported by Lacroix et al., introduced by the large tensile stress concentration building up in the periphery during micro pillar broadening.^[^ ^30^ ^]^ Once the electron beam is switched on traces of shear localization vanish immediately (Figure 3b). This indicates that even low dose electron irradiation is capable to stabilize homogeneous plasticity in amorphous silica. The peripheral radial cracks observed in beam‐off condition vanish completely. Instead, the micropillar exhibits a single big crack, usually located on the (back)side. This crack is likely due to inhomogeneous irradiation, respectively the complex interaction volume. Especially for small electron beam densities, it is likely that the interaction volume (i.e., electron range) does not cover the whole micro pillar volume. Moreover, the increasing shadowing by the indenter tip during the compression test may influence the electron range.

The electron beam intensity is first altered by increasing the acceleration voltage from 5 to 20 kV while maintaining the electron dose. As a consequence, a further softening with increasing acceleration voltage can be observed in the stress‐strain curve (Figure 2a), resulting in a yield strength reduction to roughly 6.4 GPa in the case of 20 kV (Figure 2c). At this acceleration voltage the electron beam intensity is now raised by increasing the electron dose, i.e., the electron beam current (density). The result is an even more pronounced softening visible as flattening of the stress‐strain response (Figure 2b). With increasing electron beam intensity plastic deformation becomes more and more stable. Consequently, the internal abortion criterion, given by a critical displacement rate, no longer applies. Hence, the compression experiment keeps going. In those cases, the stress increases significantly with progressing compression after an initial curve flattening when passing the yield strength. This behavior can be attributed to a micropillar broadening upon plastic deformation. Especially at high electron beam doses, the oxide glass becomes very ductile, so that the diameter of the micropillar becomes larger than the diameter of the flat indenter used for the experiments due to the high strains. In this case, the material is squeezed out of the contact zone like toothpaste (Figure 3c). When the contact area is increased, the force required for deformation increases. As the original surface area of the micropillar is used for stress conversion (technical stress), the apparent stress is increasing. This behavior is an artifact of simplification for stress conversion and is negligible at this point since the yield stress is analyzed in this study only. The yielding point exhibits a logarithmic dependency on the electron beam current density, as presented in Figure 2c. For the highest electron beam intensity, a yield strength as low as 300 MPa has been determined, which corresponds to an impressive yield strength reduction of up to 95%. This reduction is even larger than the electron irradiation‐induced drop from 8 to 10 GPa to ≈2–2.5 GPa reported by Zheng and Mackovic for nanoscale amorphous silica.^[^ ^4^, ^22^ ^]^ The literature data is added as open symbols to Figure 2c and exhibits an offset towards higher electron beam current densities compared to data from the present study. Yet, such a comparison is not straightforward as also the time (i.e., scan speed) plays a crucial role and also the computing path for the current density might differ.

## 3 Thermally Induced Softening of amorphous silica

**Figure 4:**
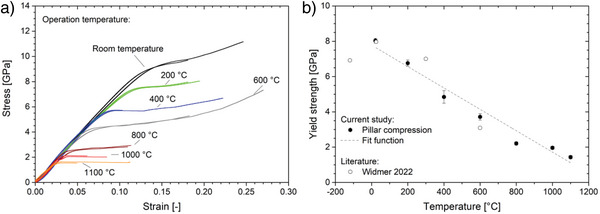
a) The stress as a function of the strain upon micropillar compression for various operation temperatures. b) The yield strength shows a linear decay with increasing electron beam current density. Literature data is shown as open symbols.^[^ ^31^ ^]^

In oxide glasses, the contribution of viscous flow to the mechanical response is usually correlated with the temperature where *T*_g_ (≈ 1200 °C) defines the point above which pure viscous flow is present. Therefore, the mechanic response of amorphous silica at elevated temperatures is expected to provide useful information for interpretation and correlation of the previous findings for electron irradiation. Widmer et al. recently studied the temperature‐dependent plasticity of micron‐scale amorphous silica in a temperature range from −120 °C to 600 °C by micropillar compression.^[^ ^31^ ^]^ The present study extends this range to 1100 °C, a temperature slightly below *T*_g_. The stress‐strain curves for various temperatures are shown in **Figure** **4**a. As upon electron irradiation, the onset of yielding is continuously decreasing with increasing temperature. Here, the yield strength exhibits a linear dependency on temperature and a remarkable conformity with the dataset obtained by Widmer and coworkers (Figure 4b).^[^ ^31^ ^]^

**Figure 5:**
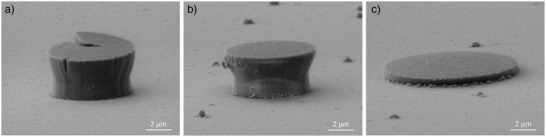
Micro pillar condition after the compression experiment at a) 200 °C as well as b) and c) 1100 °C, aborted at different strains.

A glance at the compressed micro pillar reveals plasticity to be limited by brittle fracture for temperatures below 200 °C (**Figure** **5**a). Peripheral radial cracks were observed for the beam‐off condition of the electron irradiation study. Traces of shear localization, however, were not observed. At 400 °C and beyond the mechanic response turns into a fully plastic behavior (Figure 5b). This finding is in good accordance with literature where the corresponding transition has been reported to occur between 300 and 600 °C.^[^ ^31^ ^]^ From this point on the material is likely to be too ductile to build up tensile stresses to reach the cleavage fracture limit. As a consequence, the micropillar can be compressed to a micro pancake without traces of fracture, as shown in Figure 5c for the compression at 1100 °C. It is noticeable that plasticity concentrates at the upper section of the micropillar at early stage of deformation (Figure 5a,b). This behavior contrasts with the plasticity distribution during the electron irradiation study which appears to be more homogeneously distributed throughout the whole micropillar (Figure 3b).

## 4 Discussion

**Figure 6:**
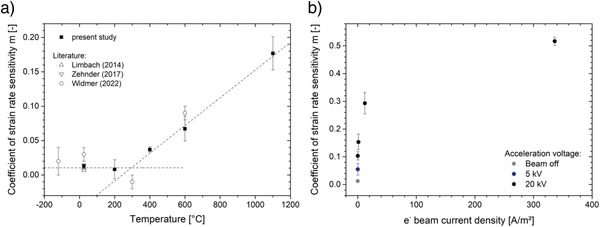
The development of the coefficient of strain rate sensitivity m upon a) annealing and b) under electron irradiation. The intersection of the dashed lines indicates the ductile‐to‐brittle transition temperature in a). Literature data is shown as open symbols.^[^ ^31^, ^34^, ^35^ ^]^

The brittle or ductile nature of vitreous silica is strongly linked to the viscosity of the material. The yield strength with its continuous decrease upon electron irradiation or annealing is not capable to define the point of brittle to ductile transition. In contrast, the strain rate sensitivity is known to change upon brittle to ductile transition.^[^ ^32^ ^]^ Superplastic flow for instance is known to stabilize in materials with high strain rate sensitivity.^[^ ^14^, ^33^ ^]^ Hence, the strain rate sensitivity has been analyzed based on a repetition of the previous study using a five times larger displacement rate. A coefficient of strain rate sensitivity *m* of 0.013 ± 0.003 has been determined under ambient conditions at room temperature, a value in great accordance with the literature.^[^ ^31^, ^34^, ^35^ ^]^ The progress of *m* upon heating is shown in **Figure** **6**a. The coefficient *m* remains constant until 200°C followed by a linear increase from 400 °C on forward. Hence, the transition from localized to homogeneous plasticity is indicated to take place between 200 and 400 °C which agrees well with the observed fracture pattern presented in Figure 5 and the study by Widmer and coworkers.^[^ ^31^ ^]^ The intersection of the line fits for each region indicates a ductile‐to‐brittle transition temperature close to 300 °C.

Under electron irradiation, the coefficient of strain rate sensitivity increases immediately (Figure 6b) indicating the transition from localized to homogeneous plasticity to occur right when the electron beam is turned on. Therefore, traces of shear localization vanish once the electron beam is running, and a homogeneous flow pattern is observed instead (Figure 3). Even moderate beam configurations (5 to 20 kV with ≈0.1 A m^−^^2^) result in *m* values ranging from 0.06 to 0.15, respectively. It is worth to point out that this range of moderate electron irradiation already resembles the m increase upon the whole annealing study of up a temperature of 1100 °C. Under higher electron irradiation the strain rate sensitivity enters a region of *m* > 0.3, which has been the threshold to define superplastic material behavior for conventional metallic alloys for a long time.^[^ ^36^ ^]^ Under maximum electron irradiation, an *m* value as high as 0.51 has been observed.

**Figure 7:**
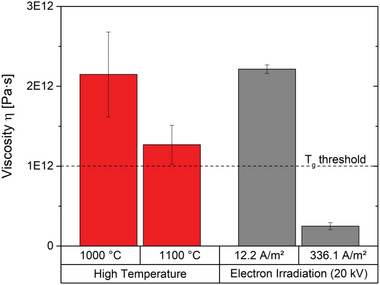
The viscosity for the two extreme conditions of each study, high temperature and electron irradiation. The threshold viscosity used to define *T*_g_ is sketched as dashed line.

It is well known that pure viscous material flow occurs in glasses at temperatures above the glass transition temperature *T*_g_ on a typical time scale of glass processing.^[^ ^11^ ^]^ For amorphous silica the transition range is starting at temperatures around 1200 °C where the threshold viscosity *η*  =  10^12^ Pa∙s is passed.^[^ ^8^, ^9^, ^10^, ^11^ ^]^ The change in stress over time allows *η* to be estimated. As the presence of pure viscous flow is questionable for low temperature as well as low electron irradiation dose, a rheology‐based model considering elastic and viscous parts of deformation has been used to assess *η*.^[^ ^37^, ^38^ ^]^ For this purpose the stress‐time curve has been fitted for the two extrema of the high‐temperature study and the electron irradiation study each. **Figure** **7** visualizes that for both 1000 °C and 1100 °C *η* does not fall below the threshold value of 10^12^ Pas, as expected for temperatures slightly below *T*_g_. In contrast, for the highest electron irradiation dose used in the present study a *η* value as low as 2.5 × 10^11^ Pas has been determined. This observation indicates that the severe electron irradiation has introduced a glass viscosity which is normally found at temperatures above *T*_g_ only. On the nanoscale electron beam induced viscosity values in the range of 10^11^ Pas have been reported for alkali‐borosilicate glasses irradiated in TEM.^[^ ^39^ ^]^

Amorphous silica exhibits the highest viscosity among any known glass‐forming system which is linked to the low flexibility of the (SiO_4_)^4−^ tetrahedra network.^[^ ^10^, ^18^ ^]^ As described by the topological constraint theory the network flexibility, i.e., viscosity, strongly depends on the atomic degrees of freedom.^[^ ^18^, ^19^ ^]^ At high temperatures siloxane bonds can be broken by the high thermal energy within the system, which increases the flexibility in the (SiO_4_)^4−^ tetrahedra network and decreases the strength and viscosity of the system has been observed in the present study.^[^ ^18^, ^19^ ^]^ At low temperatures, in contrast, the only atomic degrees of freedom arise from intrinsic network defects or trace dopants, which makes the viscosity highly susceptible to any defect source.^[^ ^18^, ^40^ ^]^ Hence, even small numbers of defects have a significant effect of the flexibility of the former stiff silica network, which is also why for instance glasses with high network modifier content exhibit a lower viscosity compared to pure amorphous silica.^[^ ^10^, ^18^ ^]^ The vitreous nature of amorphous silica provokes a broad bond angle distribution resulting in bond energy variations.^[^ ^41^, ^42^, ^43^ ^]^ Assuming that electrons accelerated with 20 kV and less create similar defects as thermal treatments, namely the formation of dangling bonds, it is conceivable to assume that a thermal treatment first affects the weakest bonds within the material, whereas the irradiation with kV accelerated electrons may affect all bonds within the glass directly. This and the high susceptibility of amorphous silica to defects make the irradiation with energetic beams a very effective tool to trigger low viscosity and “superplasticity” as it has been observed in the present study.^[^ ^4^, ^18^, ^21^, ^28^ ^]^

**Figure 8:**
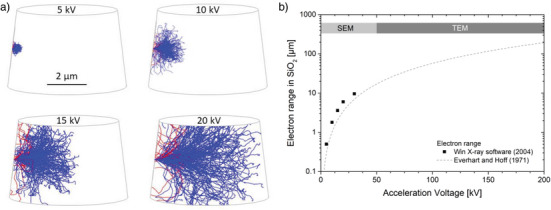
a) The electron trajectories inside the micropillar for the acceleration voltages used in the present study as calculated by Win X‐ray software Monte Carlo simulations.^[^ ^44^ ^]^ b) The electron range as a function of acceleration voltage according to win X‐ray software and a model proposed by Everhart and Hoff.^[^ ^45^ ^]^

According to Kang et al., the viscoplastic deformation of amorphous silica strongly depends on the electron range, i.e., the volume of the region where electron‐matter interaction occurs.^[^ ^26^ ^]^ Hence, the electron range has been analyzed for different acceleration voltages using the Win X‐ray Monte Carlo software.^[^ ^44^ ^]^ The resulting electron trajectories are visualized in **Figure** **8**a with the micropillar geometry sketched around them. The electron interaction volume grows with increasing acceleration voltage (by maintaining the overall shape) and covers with ≈6 µm in size almost the full micropillar volume in case of an acceleration voltage of 20 kV (please note that trajectories leaving the pillar have been truncated for better visibility). At low voltages in contrast, the electron interaction volume is pretty small compared to the micropillar volume, which explains the nearly identical mechanic response observed for 5 and 10 kV (Figure 2a). This observation agrees well with Kang's findings on amorphous silica nanospheres, who found the electron beam‐induced defect generation to be most efficient for beam parameters where the electron interaction volume is of comparable size to the amorphous silica nanospheres.^[^ ^26^ ^]^ In other words, the electron acceleration voltage defines the electron range in the material and must be selected according to the test volume for a most efficient electron distribution. It can therefore be assumed that experiments under acceleration voltages in the 200 kV region as reached in TEM or modern electron beam welding or sintering machines may allow for viscous flow in test volumes of several ten or hundred micrometers in size (Figure 8b). Once the electron interaction volume covers the full test volume, the beam dose controls the quantity of irradiation defects. With increasing defect concentration, the viscosity and thus the yield stress is significantly reduced and a high ductility is achieved (Figure 2).^[^ ^39^ ^]^ It is worth to point out that electron beam densities ranging from 1.2 to 12 A m^−^^2^, which are easily reached in any SEM, render a viscous flow usually observed thermally induced at temperatures of 600°C and beyond. It has been stated in the literature that this observation cannot be correlated to electron beam heating.^[^ ^4^, ^26^ ^]^ Nevertheless, the electron beam heating for the highest irradiation dose applied in this study has roughly been estimated to *ΔT* ≈ 32 K for an electron range of 6 µm as determined earlier.^[^ ^23^, ^46^ ^]^ This value is too low to attribute the observed softening to thermal influences especially as this value is expected to be even lower in SEM scanning mode.^[^ ^23^ ^]^ Zheng and Kang independently report a temperature rise Δ*T* below 1 K for volumes that correspond to the electron range in size.^[^ ^4^, ^26^ ^]^ It is worth noting that the 32 K should not be considered an upper limit, as things can be completely different for fully focused spots and high accelerating voltages as used in TEM.^[^ ^23^ ^]^

## 5 Conclusion

In the present study viscous flow of micron‐scale amorphous silica has been observed at room temperature as a consequence of irradiation with energetic electron beams. It has been found that on the local scale already a moderate electron irradiation resembles a mechanical state corresponding to high‐temperature properties in the range of 600—1000 °C. Under intense electron irradiation a viscosity in the range of 10^11^ Pas has been determined, indicating that T_g_ has even been passed. An impressive yield strength reduction of 95 % from 9 GPa to a value as low as 300 MPa has been determined. Those results demonstrate that electron beam‐induced superplasticity is not only limited to nanoscale geometries, for which it has been reported so far. The electron interaction volume is defined by the acceleration voltage while the defect concentration scales with the electron beam current density. The electron irradiation‐induced transition from brittle to ductile behavior occurs immediately when the electron beam is switched on whereas the thermally induced brittle to ductile transition takes place at a temperature ≈300 °C. The change in the coefficient of strain rate sensitivity *m* with T or electron beam was found to be a well‐suited indicator for this transition to take place.

Electron irradiation‐induced viscous flow offers a great potential for micro‐ and nanoscale engineering of oxide glasses, a demand widely pointed out in recent studies within the glass community.^[^ ^4^, ^31^, ^47^ ^]^ It is likely to be scalable with acceleration voltage making also larger micron‐scaled test volumes accessible. Moreover, it is expected that electron beam‐assisted shaping will not be limited to amorphous silica, as electron beam effects have already been observed in other covalent materials.^[^ ^29^, ^39^, ^48^ ^]^ Electron beam engineering is already established for a variety of materials and may have a great future in glass processing ahead of it.^[^ ^49^, ^50^ ^]^

However, electron irradiation effects can also appear as side effects and potential source of errors, especially in the context of small‐scale mechanical characterization performed in situ inside SEM. Due to their sensitivity to variations in the plastic flow behavior small scale approaches to asses fracture toughness may be particularly vulnerable to such influences of irradiation.^[^ ^51^ ^]^ Moreover, the FIB milling which is often involved in small scale sample preparation may induce additional structural modifications.^[^ ^52^, ^53^, ^54^ ^]^

## 6 Experimental Section

### Micropillar Fabrication

A commercial 500 µm thick and 100 mm diameter fused silica wafer was used as a substrate for micro pillar fabrication. The micropillars were processed using a lithography‐based deep reactive ion etching (DRIE) technique similar to the study by Ramachandramoorthy and coworkers.^[^ ^27^, ^55^ ^]^ The micropillars exhibit a final geometry with a pillar radius of 2.67 ± 0.06 µm at the top and 3.1 ± 0.12 µm at the bottom. The height corresponds to roughly 4.5 µm and a taper angle in the range of 6° was measured.

### Micropillar Compression

Nanoindentation testing was performed using the ultra‐high temperature InSEM nanoindentation system by KLA as presented by Minnert and coworkers.^[^ ^56^ ^]^ The indentation system is equipped with an InForce 1000 actuator with a 1 N load capacity. It is mounted inside a Tescan Vega 3 scanning electron microscope (SEM) equipped with a secondary electron (SE) detector and a water‐cooled backscattered electron (BSE) detector. The integrated water‐cooled BSE detector enables positioning and in‐situ experiment imaging up to the maximum operation temperature of 1100 °C by simultaneously shielding the electron column against heat irradiation.

The nanoindentation experiments were performed in a constant displacement rate mode with a velocity of 10 nm s^−1^. A velocity of 11 nm s^−1^ was set as abortion criterion to quickly stop the test upon any kind of instability (i.e., fracture). The micro pillar compression experiments were conducted with diamond flat punch indenter geometry (Synton‐MDP, Switzerland) with a punch diameter of 7 µm and 20 µm for the electron irradiation study and the high‐temperature study, respectively. Less shading is expected for the small flat punch geometry, so this indenter tip is well suited for the irradiation study whereas the bigger flat punch indenter is preferred for the high‐temperature study where positioning becomes more and more challenging with increasing operation temperature. The different indenter geometries are also responsible for the shape difference of the fully plastic‐deformed extreme cases comparing Figure 3c and Figure 5c.

### Electron Beam Dose Variation

The electron beam intensity was altered via first increasing the SEM acceleration voltage from 5 kV in steps of 5 kV to 20 kV while keeping the beam intensity constant. Then, the acceleration voltage was kept constant at 20 kV while the beam intensity in the SEM control software was increased from 6, over 10 and 14 to finally 20. The micropillar compression experiments were monitored under a 20 kx magnification, a magnification where the micropillar covers almost 50 % of the SEM image (compare Figure 1) using a scan speed of 0.32 µs per pixel. The incident electron beam current was determined by shooting with similar magnification into the faraday cup. Doing so electron beam currents ranging from 7 pA to 27.5 nA were measured. A detailed overview of the beam parameter is provided in **Table** **1**. The electron beam dose was roughly estimated by referring the electron beam current to the monitored area (768 × 576 pixels with a pixel size of ≈13.6 nm), corresponding to ≈82 µm^2^. The positioning procedure for the micropillar tested under beam‐off condition was performed under the smallest beam intensity to reduce the risk of irreversible material alteration upon irradiation.

**Table 1:** Beam parameter used for electron irradiation experiment

Acceleration voltage [kV]	Beam intensity	Beam current [pA]	Spot size [nm]	Beam density [A m^−^^2^]
5	6	7	84	0.09
10	6	9	61	0.11
15	6	8	51	0.10
20	6	11	47	0.13
20	10	100	163	1.22
20	14	1000	411	12.22
20	20	27500	1494	336.10

### Data Conversion

The load displacement data is transferred into stress strain data by first referring the load F to the initial top surface area A of the micropillar to obtain the stress *σ*:

(1) $$\sigma =\frac{F}{A}=\frac{F}{\pi \cdot r^{2}}$$

A simple estimate of strain can be derived by referring the displacement signal, i.e., penetration depth h, to the initial micro pillar height *h*_0_:

(2) $$\varepsilon =\frac{h}{h_{0}}$$

This strain value, however, is strongly affected by the machine compliance. Hence, a too small elastic modulus would be derived from the linear elastic segment of the stress‐strain curve. As we know the elastic slope should match the literature elastic modulus of fused silica (70 GPa), this information can be used to correct the *ε* data. A correction factor close to 0.5 was determined at room temperature without electron beam irradiation. This value was used for the analysis of all micropillar compression tests even though the machine compliance as well as the elastic modulus of fused silica are expected to change with increasing temperature.^[^ ^56^, ^57^, ^58^ ^]^ As the scope focus of this study lies on the yield stress, we accept this possible inaccuracy in strain. Due to the local impact during the electron beam irradiation study, changes in machine compliance are not expected. Changes in the elastic slope can, therefore, be directly linked to an electron irradiation effect on the materials mechanic response.

Even though the softening upon electron irradiation and during heating is obvious, the smooth transition from elastic to elastic‐plastic region makes an objective determination of the yielding point difficult. Therefore, the for metallic materials well established concept of R_p0.2_ has been applied to the stress‐strain curves of fused silica. The yield stress is determined from the intersection of the stress‐strain curve with a line parallel to the initial elastic slope shifted by 0.2 % strain.

### Strain Rate Sensitivity

The micropillar compression experiments were repeated with a velocity of 50 nm s^−1^ and an abortion criterion of 55 nm s^−1^ to study strain rate sensitivity. The coefficient of strain rate sensitivity was determined using Equation 3.^[^ ^59^, ^60^ ^]^

(3) $$m=\frac{d\left(\ln\sigma \right)}{d\left(\ln\dot{\varepsilon }\right)}$$

### Viscosity

The viscosity *η* has been assessed by fitting the loading part of the stress‐time data with a rheology‐based model according to Equation 4.^[^ ^37^, ^38^ ^]^

(4) $$\sigma \left(t\right)=\dot{\varepsilon }\cdot \eta \cdot \left[1-\exp\left(\frac{E_{M}}{\eta }\cdot t\right)\right]+E_{H}\cdot \dot{\varepsilon }\cdot t$$

It is worth to mention that an accurate determination of *η* was beyond the scope of this study and requires an adjusted experimental approach. The values presented in Figure 7 should be considered as a rough estimate rather than accurate absolute values.

## Conflict of Interest

The authors declare no conflict of interest.

## Data Availability

The data that support the findings of this study are available from the corresponding author upon reasonable request.
